# Experience of the Postoperative Intensive Care Treatment of Stanford Type A Aortic Dissection

**DOI:** 10.1155/2023/4191277

**Published:** 2023-01-10

**Authors:** Lei Bai, Lijuan Ge, Yujing Zhang, Mingliang Li, Bo Jiang, Yanyan Song

**Affiliations:** ^1^Department of Cardiovascular Surgery, General Hospital of Ningxia Medical University, Yinchuan 750004, China; ^2^Department of Pediatrics, General Hospital of Ningxia Medical University, Yinchuan 750004, China

## Abstract

**Objective:**

To summarize the experience of the postoperative intensive care treatment of Stanford type A aortic dissection (STAAD) following Sun's procedure.

**Methods:**

A total of 124 patients with STAAD who underwent Sun's procedure from January 2014 to December 2021 at the General Hospital of Ningxia Medical University were retrospectively analyzed. All patients were admitted to the cardiac surgery intensive care unit (ICU) after surgery. According to the perioperative characteristics of the patients with STAAD, intensive care treatment was given to actively prevent the occurrence of postoperative complications.

**Results:**

In all the cases enrolled in this study, the causes of aortic dissection comprised hypertension (105 cases), trauma (six cases), Marfan's syndrome (six cases), and aorto-arteritis (seven cases). The history of past illnesses comprised hypertension (105 cases), coronary disease (25 cases), diabetes mellitus (16 cases), and chronic obstructive pulmonary disease (six cases). There were some preoperative complications, such as cardiac insufficiency, acute liver insufficiency, acute renal insufficiency, pleural effusion, pericardial effusion, pulmonary infection, lower limb ischemia, mesenteric arterial embolism, and digestive tract hemorrhage. The average cardiopulmonary bypass time was 186 ± 32.1 min, the aortic clamp time was 74 ± 12.8 min, the deep hypothermic circulatory arrest time was 21 ± 2.6 min, and the mechanical ventilation time was 34 ± 2.8 h. The average ICU and hospital residence times were 7 ± 1.6 days and 12 ± 3.6 days, respectively. Postoperative complications comprised hypoxemia (34 cases), pulmonary infections (22 cases), tracheostomy (four cases), cerebral hemorrhage (four cases), cerebral infarction (four cases), transient delirium (eight cases), secondary thoracotomies due to bleeding (two cases), alimentary tract hemorrhage (eight cases), and acute renal insufficiency (38 cases). There was no occurrence of hoarseness or chylothorax. There were 15 cases of death, and the total mortality rate was 12.1%. In four cases, the cause of death was one postoperative complication (3.2%), and in 11 cases, the cause of death was multiple postoperative complications (8.9%). The other patients were discharged from the hospital with a good prognosis for full recovery.

**Conclusion:**

Postoperative intensive care treatment was an important part of the successful surgical treatment of STAAD.

## 1. Introduction

Aortic dissection (AD), also known as an aortic dissection aneurysm, is a serious aortic disease in which blood in the lumen of the aorta enters the middle layer of the arterial wall through the endothelial rupture after tearing. It then forms an entrapment hematoma, which expands in the direction of the long axis of the vessel and forms pathological changes in the true and false lumens of the artery. Stanford type A aortic dissection (STAAD) is an aggressive and critical condition in cardiovascular surgery with a high mortality rate if not treated surgically in time [1–3]. The clinical characteristics of AD are acute onset, sudden onset of severe pain, hypertension, cardiac manifestations, ischemic symptoms of other organs or limbs, etc. If not treated in time, the mortality rate is as high as 50% within 48 h [4]. The main causes of death are the rupture of the aortic dissection aneurysm, causing thoracic, abdominal, or pericardial cavities; progressive mediastinal and retroperitoneal hemorrhages; and acute heart and renal failure [5].

Patients in the acute phase should be given intensive medical drug therapy first, regardless of whether interventional or surgical treatment is undertaken. In the case of ascending AD, especially if the aortic valve is affected or if there is fluid in the pericardium, emergency surgery is recommended. In the acute stage of descending AD, if the disease progresses rapidly and the local vessel diameter is ≥5 cm or there are vascular complications, interventional treatment with stent implantation (endoluminal isolation of arteries) should be sought. In fact, AD progresses rapidly, and the mortality rate is extremely high in patients who are not treated promptly: 33% of patients die within 24 h, and the mortality rate increases by 1%–2% per hour within 48 h. A percentage of 80% of patients die within a week, and 75% of patients die from an aortic rupture [6]. Early diagnosis and treatment of AD have a great impact on the prognosis.

Sun's procedure is the new criteria for the surgical management of complex type A AD. This procedure was started in 2003, and according to the morphological characteristics of aortic diseases in China, Professor Sun Lizhong and others from Beijing Anzhen Hospital Affiliated with Capital Medical University developed and applied a self-developed stent artificial blood vessel to develop the new aortic arch replacement and stent elephant trunk surgery [7]. This procedure is suitable for the treatment of complex AD, extensive aortic lesions involving the aortic arch, and the descending part of the arch.

Despite significant improvements in surgical techniques and management, perioperative mortality remains high. The literature reports that the postoperative mortality of acute STAAD is 9%–30% [8]. Therefore, it is important to enhance the perioperative critical care management of these patients. This study analyzes the postoperative intensive care treatment of 124 patients with STAAD who were treated with Sun's procedure from January 2014 to December 2021.

## 2. Materials and Methods

### 2.1. Clinical Materials

The inclusion criteria were as follows: (1) preoperative diagnosis of STAAD confirmed by cardiac ultrasound and thoracoabdominal aortic computed tomography angiography (CTA) with clinical symptoms and signs; (2) non-reoperative AD surgery; (3) surgical indications and agreement to accept surgical treatment. The exclusion criteria were as follows: (1) preoperative and intraoperative death; (2) rejection of surgical treatment; (3) no surgical indications; (4) other heart surgeries were performed at the same time; (5) one-stop hybrid procedures. The primary endpoint was postoperative death, and the secondary endpoint was postoperative complications.

The patients comprised 78 men and 46 women, with an age range of 31–68 years and an average age of 49.8 ± 3.8 years. Their weight ranged from 57 to 110 kg, with an average weight of 77.8 ± 6.8 kg. The diagnosis of STAAD was confirmed for all patients using CT angiography of the aorta. The causes of AD comprised hypertension (105 cases), trauma (six cases), Marfan's syndrome (six cases), and aorto-arteritis (seven cases). The history of past illnesses comprised hypertension (105 cases), coronary disease (25 cases), diabetes mellitus (16 cases), and chronic obstructive pulmonary disease (six cases). There were some preoperative complications, such as cardiac insufficiency, acute liver insufficiency, acute renal insufficiency, pleural effusion, pericardial effusion, pulmonary infection, lower limb ischemia, mesenteric arterial embolism, and digestive tract hemorrhage. The details are illustrated in Table 1.

Cardiac color ultrasonography and CTA of the aorta were conducted in all cases to diagnose the site and extent of the lesion as well as its relationship with the surrounding organs. Indications for surgery were followed by the establishment of the surgical approach. Emergency surgery was conducted in 78 cases.

### 2.2. Preoperative Therapy

Before the operation, the patients were bedridden through sedation and analgesics. The blood pressure was actively controlled to achieve a stabilization status (the systolic blood pressure was <120 mmHg). Blood glucose was monitored together with other supportive treatments. If the ventricular rate was >100 beats/min, esmolol was administered preoperatively to maintain it through continuous pumping at <80 beats/min.

### 2.3. Surgical Methods

All patients underwent a median thoracotomy with compound intravenous anesthesia and heparinization. Conventional standby artery single pump double tube, right axillary artery cannulation (sometimes combined with femoral artery cannulation), right atrial bipolar cannulation for drainage, right superior pulmonary vein left heart drainage, and deep hypothermic circulatory arrest (DHCA) were conducted. With the establishment of extracorporeal circulation, the ascending aorta was blocked. The proximal operation was conducted first, and the treatment of the proximal aorta mainly depended on pathological changes. Based on whether the proximal dissection involved the sinus, aortic valve leaflets and valve annulus, or coronary arteries, different processing methods were selected. The nasopharyngeal temperature was induced to drop to 18°C–20°C with a rectal temperature of 25°C. The aortic arch was reconstructed with a four-branch vessel prosthesis under the circulation arrest, and an elephant trunk stent was implanted in the true lumen of the descending aorta to complete the classic Sun's procedure. During the period between the start of the circulatory arrest and the selective cerebral perfusion (flow 10–15 ml/kg/min), the left carotid artery was directly perfused.

### 2.4. Postoperative Intensive Care and Treatment

All patients were admitted to the cardiac surgery intensive care unit (ICU) for postoperative monitoring and treatment. Electrocardiogram (ECG), blood pressure, urine output, and drainage fluid (the drainage from the pericardial, mediastinal, and chest drainage tubes) were monitored. All patients were monitored for consciousness through the clinical signs and symptoms of all systems. The detected and monitored indicators included hepatic, renal, coagulation functions, routine blood tests, blood gas analyzes, lactic acid tests, and blood glucose tests. The tested indicators included ECG, chest X-ray, cranial CT scan, and cardiac ultrasonography. Postoperatively, invasive mechanical ventilation was conducted until the condition stabilized, with extubation subsequently carried out. Noninvasive ventilation was conducted on patients with hypoxemia until their conditions stabilized. Based on the clinical conditions of different systems, postoperative treatment with vasoactive drugs, dehydrating agents, hemostatic agents, and gastrointestinal mucosal protective agents was carried out. Based on the hepatic and renal functions and the chest X-ray, hepatic and renal function protection, as well as anti-infection and expectorant treatment, were conducted. Furthermore, electrolytes were supplemented, the internal environmental disorders were corrected, and enteral and parenteral nutrition support were provided. Hemodialysis was carried out in the case of renal failure.

### 2.5. Statistical Methods

The statistical analysis was performed by another blinded statistician using SPSS 19.0 software. The measurement data were expressed as mean ± standard deviation $\left(\bar{\chi }\pm s\right)$, and the *t*-test was used for statistical analysis. The countable data were expressed as percentages or rates.

## 3. Results

### 3.1. Intraoperative and Postoperative Clinical Data of Cases in the Whole Group

In all the cases enrolled in the study, the average cardiopulmonary bypass time was 186 ± 32.1 min, the aortic clamp time was 74 ± 12.8 min, the DHCA time was 21 ± 2.6 min, and the mechanical ventilation time was 34 ± 2.8 hours. The average ICU and hospital residence times were 7 ± 1.6 days and 12 ± 3.6 days, respectively. The details are illustrated in Table 2.

### 3.2. Postoperative Complications and Clinical Outcomes in the Whole Group

The postoperative complications included hypoxemia (34 cases), pulmonary infections (22 cases), tracheostomy (four cases), cerebral hemorrhage (four cases), cerebral infarction (four cases), transient delirium (four cases), secondary thoracotomy due to bleeding (two cases), alimentary tract hemorrhage (eight cases), and acute renal insufficiency (38 cases). There was no occurrence of hoarseness or chylothorax. There were 15 cases of death, and the total mortality rate was 12.1%. In four cases, the cause of death was one postoperative complication, and in 11 cases, the cause of death was multiple postoperative complications, comprising severe pulmonary infection (four cases), circulatory failure due to postoperative hemorrhage (two cases), postoperative acute renal failure leading to multiple organ failures (four cases), alimentary tract hemorrhage (three cases), and LCOS (two cases). The other patients were discharged from the hospital with a good prognosis. The details are presented in Table 3.

## 4. Discussion

Stanford type A AD is a life-threatening and critical condition with a poor prognosis, complex surgery, and a high mortality rate. In recent years, due to the increased awareness of the disease among medical staff and the advancement of diagnosis and treatment techniques, the diagnosis rate has increased significantly and the mortality rate has decreased. The recent experience of intensive care treatment after surgery for STAAD in the hospital in question is summarized in this study.

### 4.1. Respiratory System Maintenance

In acute AD, the dilated aorta compresses the airway and pleural effusion compresses the lungs, affecting the ventilation function, while the outbreak of systemic inflammatory factors causes major damage to the alveolar tissues and affects the gas exchange function [9]. The incidence rate of hypoxemia after thoracotomy for STAAD has been reported to be as high as 51% [10]. Hypoxemia refers to the lack of oxygen in the blood, with the arterial oxygen partial pressure (PaO_2_) below the normal lower limit of the corresponding age, which mainly manifests as blood oxygen partial pressure and blood oxygen saturation decline and is defined as PaO_2_/FiO_2_ ≤ 300 mmHg.

Hypoxemia is a common complication after Sun's procedure. Obesity, massive blood transfusion, the long duration of DHCA, and preoperative combined hypoxemia are independent risk factors for postoperative hypoxemia following this procedure [11]. Massive surgical injuries and prolonged mechanical ventilation also increase the incidence of ventilator-associated pneumonia. Perioperative massive transfusion of blood products and ischemia-reperfusion injury to the lungs during DHCA may also cause pulmonary impairment and can lead to postoperative hypoxemia.

The focus of the postoperative respiratory intensive care was monitoring the ventilator parameters and adopting a lung-protective ventilation strategy for patients who developed postoperative hypoxemia (defined as PaO_2_/FiO_2_ ≤ 300 mmHg) with a tidal volume of 6–8 ml/kg along with lung recruitment. The arterial blood gas was monitored through the adjustment of the ventilator parameters according to the blood gas results. The chest X-ray was reviewed daily for pulmonary imaging changes. Any sputum was examined, including in terms of volume and properties, and retained for culture testing. Respiratory care and physical therapy were critical to preventing pulmonary infections. To reduce the inflammatory response during extracorporeal circulation, high-dose methylprednisolone was administered prophylactically and continued at a low dose for 72 h postoperatively. This was to reduce the incidence of lung injury and postoperative hypoxemia. In this study, there were 34 cases of hypoxemia with an incidence rate of 27.4%, and there were 22 cases of pulmonary infection with an incidence rate of 17.7%, which is lower than the highest incidence rate reported in the literature [10–12].

The lower incidence of hypoxemia and pulmonary infection in this study was considered to be related to ICU management experience and perioperative intervention. A full understanding of the risk factors affecting postoperative pulmonary functions, eliminating these risk factors as far as possible, taking appropriate therapeutic measures if necessary, perfecting the surgery, and intensifying the postoperative treatment of all systems are essential for early extubation and for reducing the possibility of postoperative pulmonary function abnormalities. Early extubation can reduce pulmonary complications, promote early bed mobility, and shorten the length of hospital stays. The interventions used included the preoperative cessation of smoking, the treatment of various cardiopulmonary diseases, and the improvement of oxygenation and ventilation. Preoperative blood transfusion should ensure the hematocrit ≥30% to reduce the degree of hemodilution and the use of blood products. Preoperatively, cardiac and renal functions should be improved as much as possible, the duration of extracorporeal circulation and intraoperative fluid input should be reduced during surgery, and postoperative bleeding should be effectively controlled and the input of blood products should be reduced. Furthermore, short-acting postoperative sedative drugs should be used to enable the patient to awaken within a few hours after drug withdrawal to facilitate early extubation. Among all the patients, 30 recovered following treatment at a rate of 88.23%, which is higher than the level reported in the existing literature [12]. Four patients died of severe pulmonary infections.

### 4.2. Central Nervous System Maintenance

Sun's procedure is the main treatment option for STAAD. The procedure should be carried out under DHCA and may induce neurological complications, especially cerebral complications and paraplegia, which have high incidence rates. It has been reported that the prolonged duration of DHCA [13, 14], high fluctuations of intraoperative mean arterial pressure [15], massive infusion of red blood cells, renal insufficiency, and postoperative low cardiac output syndrome are the meaningful independent predictors of cerebral complications following surgery for STAAD [16].

The most common central nervous system (CNS) complications include ischemic and hypoxic cerebral injury, cerebral hemorrhage, cerebral infarction, postoperative impaired consciousness (delirium, mental abnormalities, excitement, etc.), and paraplegia [17–19]. During postoperative monitoring, close attention should be paid to the response of the CNS, as well as to consciousness, limb activity, and pathological reflexes. Early postoperative dehydration using mannitol, glycerol, fructose, or diuretics should be given to lower the cranial pressure. Furthermore, postoperative methylprednisolone should be administered to reduce inflammatory reactions, and, if necessary, medication should be given to promote the recovery of the cerebral functions and improve the brain metabolism [20]. During mechanical ventilation, hypoxemia and hypercarbia should be prevented. Blood glucose levels should be monitored and maintained within the normal range to avoid hypoglycemia or hyperglycemia. Hyperthermia must be avoided, and the body temperature should be lowered, especially the head temperature, which is an effective way to protect the nervous system.

During the operation, the right axillary artery cannulation and selective cerebral perfusion technique could effectively ensure cerebral blood perfusion, which would be effective in protecting the CNS. No sedatives were administered before the patients awakened postoperatively, and they were treated appropriately after regaining consciousness. The symptoms of the patient with paraplegia improved after a week of cerebrospinal fluid drainage, and the patient was later transferred to the rehabilitation department for further treatment. Following postoperative treatment, all patients recovered and were discharged from the hospital with good prognoses.

### 4.3. Postoperative Renal Function Maintenance

Acute renal failure following STAAD is associated with preoperative dissection involving the renal artery, impaired renal function due to long-term hypertension, intraoperative renal ischemia due to surgical injury, massive transfusion of blood products, and postoperative blood volume deficiency. Here, when postoperative renal impairment occurred, medications were administered for renal protection. If the effect was poor, early bedside hemodialysis treatment was required. In this study, there were 38 cases of acute postoperative renal insufficiency, with an incidence rate of 30.6%, and there were four cases of acute renal failure (3.2%). Through hemodialysis and drug treatment, renal functions were restored in 34 cases, and they were discharged from the hospital after recovery.

### 4.4. Treatment for Postoperative Hemorrhage

The surgery for AD was conducted under DHCA, which destroys the coagulation mechanism and platelets, resulting in intraoperative and postoperative blood leakage from the surgical wound and vascular anastomosis, increased drainage, and the inability to stop extracorporeal circulation support due to blood leakage from the anastomosis. Intraoperative and postoperative transfusion of cold precipitation and platelets, as well as the supplementation of coagulation factors, could improve the coagulation function. The application of recombinant human coagulation factor VII could improve any anastomotic blood leakage [21, 22]. During the ICU stay, the changes in drainage (the pericardial, mediastinal, and chest drainage tubes), central venous pressure, blood pressure, hematocrit, and hemoglobin were closely monitored to comprehensively determine the existence of hypovolemia and supplement the crystalloids and colloids. After the operation, it was ensured that the drainage tubes were monitored and kept unobstructed. If the drainage volume exceeded 200 ml/hour and lasted for more than 3 h, it indicated an active hemorrhage and the urgent need for a second thoracotomy to achieve hemostasis and avoid serious outcomes. In this study, there were two cases of second thoracotomies.

### 4.5. Treatment of Low Cardiac Output Syndrome

The main clinical manifestations of low cardiac output syndrome are a rapid heart rate, low or unstable blood pressure, poor peripheral circulation, and low urine output. Low cardiac output after acute STAAD is mainly associated with preoperative AD involving the aortic root and coronary artery, intraoperative myocardial ischemia-reperfusion injury, and early postoperative internal environmental disturbance. Low cardiac output syndrome not only leads to hemodynamic instability but also aggravates injuries to other organs. The effective prevention and treatment of low cardiac output is the key to successful early postoperative treatment. Inotropic drugs such as dopamine, dobutamine, and milrinone should be administered. If the circulation is unstable, adrenaline might be considered an adjunct, while nitroglycerin could dilate the coronary arteries to increase the myocardial blood supply and improve microcirculation. The duration of mechanical ventilation should be extended appropriately, depending on the circulation.

## 5. Conclusion

Sun's procedure is a classic for the treatment of acute STAAD and is safe and effective. However, the procedure is complex, with a long, high-risk operation, and the postoperative changes may involve various systems. The postoperative intensive care treatment of STAAD is an important part of its successful surgical treatment, especially in maintaining the CNS, respiration, circulation, coagulation, and hepatic and renal functions. For the successful surgical treatment of STAAD, it is critical to maintain the stability of the internal environment and prevent and control any postoperative complications.

The clinic needs to pay sufficient attention to the perioperative management of patients with AD to reduce the risk of various complications and must intervene in a timely manner for any that have occurred. Preventive and therapeutic measures include improving the surgical techniques, shortening the duration of extracorporeal circulation, applying hormones and anti-inflammatory agents, actively managing low cardiac output, hypoxemia, and acute renal impairment, maintaining cardiac functions, controlling any bleeding, enhancing gastrointestinal management, and preventing infection.

## Figures and Tables

**Table 1:** Preoperative clinical data of the patients $\left(\bar{\chi }\pm s\right)$ [*n* (%)].

Item	Clinical data
Gender (male/female)	(78/46)
Age (year old)	49.8 ± 3.8
Weight (kg)	77.8 ± 6.8
Cause of the aortic dissection	
Hypertension	105 (84.7)
Trauma	6 (4.83)
Marfan syndrome	6 (4.83)
Aorto-arteritis	7 (5.64)
History of past illness	
Hypertension	105 (84.7)
Coronary disease	25 (20.2)
Diabetes mellitus	16 (12.9)
COPD	6 (4.83)
Preoperative complications	
Cardiac insufficiency	6 (4.83)
Acute liver insufficiency	11 (8.87)
Acute renal insufficiency	38 (30.6)
Pleural effusion	15 (12.1)
Pericardial effusion	10 (8.06)
Pulmonary infection	7 (5.64)
Lower limb ischemia	5 (4.03)
Mesenteric arterial embolism	4 (3.22)
Digestive tract hemorrhage	4 (3.22)

**Table 2:** Intraoperative and postoperative clinical data of the patients $\left(\bar{\chi }\pm s\right)$.

Item	Clinical data (*n* = 124)
Extracorporeal circulation (min)	186 ± 32.1
Aortic clamp (min)	74 ± 12.8
DHCA (min)	21 ± 2.6
Mechanical ventilation (h)	34 ± 2.8
Dwell time of CSICU (d)	7 ± 1.6
Use of antimicrobial agents (d)	8.13 ± 3.25
Hospital stays (d)	12 ± 3.6

**Table 3:** Postoperative complications and clinical outcomes of the patients (*n*, %).

Item	Clinical data (*n* = 124)	Clinical outcomes	
		Recure	Death in hospital
Hypoxemia	34 (27.4)	34	0
Tracheotomy	4 (3.2)	4	0
Cerebral hemorrhage	4 (3.2)	4	0
Cerebral infarction	4 (3.2)	4	0
Transient delirium	8 (6.4)	8	0
Secondary thoracotomy (hemorrhage)	2 (1.6)	2	2
Acute liver insufficiency	2 (1.6)	2	0
Acute renal insufficiency	38 (30.6)	34	4
Pleural effusion	48 (38.7)	48	0
Pulmonary infections	22 (17.7)	18	4
Alimentary tract hemorrhage	8 (6.4)	5	3
LCOS	2 (1.6)	0	2
Death	15 (12.1)		
Multiple complications	88 (71.0)		11
One complication	36 (29.0)		4

## Data Availability

Materials described in the manuscript, including all relevant raw data, will be freely available to any scientist wishing to use them for noncommercial purposes, without breaching participant confidentiality.
